# Lumpy Skin Disease (LSD) in Yak (*Bos grunniens*): An Evidence of Species Spillover from Cattle in India

**DOI:** 10.3390/microorganisms11122823

**Published:** 2023-11-21

**Authors:** Gundallahalli Bayyappa Manjunatha Reddy, Sai Mounica Pabbineedi, Sudeep Nagaraj, Shraddha Bijalwan, Sunil Tadakod, Zeruiah Bhutia, Diki Palmu, Seema Rai, Pempa Doma Bhutia, Pem Tshering Bhutia, Emila Shenga, Baldev Raj Gulati

**Affiliations:** 1 ICAR-National Institute of Veterinary Epidemiology and Disease Informatics, Bengaluru 560064, India; mounica20sai@gmail.com (S.M.P.); sudeepcellculture@gmail.com (S.N.); shraddhabijalwan1998@gmail.com (S.B.); sunilnivedi@gmail.com (S.T.); brgulati@gmail.com (B.R.G.); 2 Animal Husbandry and Veterinary Services Department, Tadong, Sikkim 791109, India; zeruiahbhutia@gmail.com (Z.B.); dikipalmu17@yahoo.co.in (D.P.); seemaraivet87@gmail.com (S.R.); drpempadoma@gmail.com (P.D.B.); fmdcellskm@gmail.com (P.T.B.); sernya2014@gmail.com (E.S.)

**Keywords:** cattle, India, lumpy skin disease virus, phylogenetic analysis, spillover, yak

## Abstract

Lumpy skin disease (LSD), caused by the lumpy skin disease virus (LSDV), is a global concern that affects cattle and buffalo. Recently, the disease has been reported in new species such as the Indian Gazelle, Camel, Banteng, Gaur, and Giraffe from various parts of the world. This report provides an insight into the occurrence of LSD in Yak from Sikkim, a North-Eastern state of India. During the investigation, both cattle and yak exhibited typical clinical signs of LSD, including skin nodular lesions. The morbidity, mortality, and case fatality rates for cattle were 9.08%, 1.84%, and 20.24%, respectively. Similarly, the morbidity, mortality, and case fatality rates in yak were 7.57%, 1.24%, and 16.33%, respectively. The virus isolation and amplification of LSDV-specific genes confirmed the presence of LSDV in cattle, yak, and vectors. Further, demonstrated antibodies in randomly collected sera from naïve and unvaccinated cattle and yak using indirect Enzyme Linked Immuno-sorbent Assay (iELISA) and Serum Neutralisation test (SNT) from this region. Sequencing and phylogenetic analysis of *P32*, *GPCR*, and *RPO30* genes revealed that the virus isolated from both species was 100% identical to each other and also closely related to the field LSDV isolates circulating in the Indian subcontinent. The study highlighted the emergence of LSDV in unconventional hosts and underscored the need to include other bovine species in national disease control programs, encompassing disease surveillance initiatives.

## 1. Introduction

Lumpy skin disease (LSD) is a significant vector-borne and re-emerging transboundary disease that affects cattle, and buffaloes, and poses a serious threat to the farming sector. Historically, LSD was first reported in Zambia in 1929 and was confined to sub-Saharan regions until 1986 [1]. Since then, the disease has spread to Middle Eastern countries and neighbouring regions. Recently, the outbreaks were reported from various Asian countries, including India [2], Bangladesh [3], China [4], Nepal [5], Myanmar [6], and Pakistan [7]. Considering the transboundary spread and economic impacts caused by the disease, the World Organisation for Animal Health (WOAH) has categorised LSD as a notifiable disease [1]. India, consisting of the highest bovine population in the world (302.35 million), has been significantly affected by the LSD outbreaks. The disease was first reported in Odisha in August 2019 [2] and spread to neighbouring states such as Andhra Pradesh, Madhya Pradesh, Kerala, and Assam during 2020–2021. Subsequently, it spread to the North-Western states: Gujarat, Rajasthan, Punjab, Haryana, and Himachal Pradesh and the Southern states: Karnataka, Tamil Nadu, Kerala, and Telangana by the end of 2022 [8]. Notably, during the outbreak that occurred in 2022, where 1,55,000 cattle succumbed to LSD [9] as compared to the 2019 outbreaks, where no mortality was reported [2]. Further, it was estimated an economic loss of USD 2217.26 million in India due to LSD outbreaks in bovines [10].

LSD is caused by the Lumpy skin disease virus (LSDV), a member of the genus Capripoxvirus, subfamily Chordopoxvirinae, and family Poxviridae. It is an enveloped virus consisting of a distinctive dumbbell-shaped core and lateral bodies. The genome comprises 151 kbp of double-stranded DNA with inverted terminal repeats of approximately 2.4 kbp. The central portion of the DNA encodes 156 open reading frames (ORFs) annotated as putative genes and consists of 146 conserved genes necessary for replication, transcription, and assembly [11]. Further, the other members of the capripoxvirus genus, the Goatpox virus (GTV) and Sheeppox virus (SPV) share 97% similarity with the genome of LSDV and are attributed to the indistinguishable nature of the members of the genus Capripoxvirus in serology [1,11].

In general, LSDV is considered to be mainly transmitted by the arthropod vectors, both direct and indirect transmission is possible. The vector-associated mechanical transmission is due to biting insects like Stomoxys, House fly, Culex, and Aedes mosquito, tsetse flies, biting midges like Culicoides, and ticks such as Amblyomma, Rhipicephalus, and Hyalomma [1]. There is also a possibility of transstadial and transovarial transmissions through ticks [12,13]. The virus is primarily spread locally by vectors, but long-distance dissemination can occur through cattle trade, contributing to the re-emergence of the disease in previously disease-free areas [1].

LSD follows an incubation period spanning from 4 days to 4 weeks and clinically, manifests in acute, subacute, and chronic forms, affecting cattle of all age groups and breeds [1]. However, among cattle, *Bos taurus* is more susceptible to the disease compared to *Bos indicus* and African water buffalo are clinically susceptible to LSD [14]. The clinical signs typically begin with a fever (40 °C to 41.5 °C), accompanied by increased oro-nasal, pharyngeal, and lachrymal secretions, swelling of superficial lymph nodes, loss of appetite, reduced milk production, and depression. The skin nodules begin to emerge within one to two days post-infection, inducing severe pain and discomfort in the affected animals. As the disease progresses, 2–3 weeks, the nodules become necrotic and develop into characteristic “Sit-fast”, sequestrum of necrotic material which eventually slough off resulting in cavities that can potentially lead to bacterial infection [15]. Moreover, bulls may experience temporary or permanent infertility and excrete the virus in their semen for an extended period [2]. The average morbidity rate for LSD infection varies from 5% to 45%, sometimes it may go up to 100%, whereas the mortality rate is usually below 10% [15] resulting in significant economic consequences due to direct and indirect losses, attributed to substantial reduction in milk production, weight loss, infertility, reduced hide quality, chronic debilitation, abortion, and mortality [15].

Globally, LSD has been reported in unnatural hosts such as the Indian Gazelle (*Gazella bennettii)* [16], Camel [17], Banteng *(Bos javanicus)*, Gaur *(Bos gaurus)* [18], and Giraffe [19]. This study reports the first instance of LSD in Yak (*Bos grunniens*) in India, which is closely related to the cattle species, the main host for LSDV.

## 2. Materials and Methods

### 2.1. Ethics Statement

The study involved the collection of biological samples from Cattle and Yak. The samples of skin scabs, nasal swabs, and blood (5 mL each) were collected by the veterinarian following the standard protocol without using anaesthesia. The Animal Husbandry and Veterinary Services Department, Sikkim state, India had granted the permission for collection of the samples. Also, due consent was obtained from the animal owner before the collection of the samples.

### 2.2. Cells

African green monkey kidney cells (Vero cell line) were utilised for virus isolation, while Madin Darby Bovine Kidney cells (MDBK) were used for the serum neutralisation test (SNT). These cell lines were maintained in Minimum Essential Medium (MEM) supplemented with antibiotics and 10% Fetal Bovine Serum (FBS) at the ICAR—National Institute of Veterinary Epidemiology and Disease Informatics (NIVEDI), Bengaluru, Karnataka, India.

### 2.3. Virus and Serum Controls

A field isolate, LSDV/Cattle/India/Chitradurga/P34, was obtained from the scab tissue of infected cattle and adapted to the MDBK cell line at ICAR-NIVEDI. This isolate was used to prepare a 100 TCID50 virus suspension for the SNT and also served as a positive control in the Polymerase Chain Reaction (PCR). For indirect Enzyme Linked Immunosorbent Assay (iELISA) and SNT, positive control sera were obtained from LSD-infected cattle, while negative control sera were sourced from cattle unaffected by LSD.

### 2.4. Study Area and Data Collection

The study covered the Sikkim state, located in the North-Eastern part of India, which shares its borders with Tibet in the North, West Bengal in the South, Nepal in the West, and Bhutan in the East. The state consists of a total cattle population of 146,746 and a yak population of 5219 (as per the 20th Livestock Census, DAHD, GOI). The details regarding the LSD outbreak in this state were collected by the Department of Animal Husbandry and Veterinary Services, Sikkim. Reportedly, the state experienced its first case of LSD in cattle in the month of December 2022, with a notable surge in outbreaks beginning in May 2023, and peaking in July 2023. A total of 15,403 cases were recorded in cattle located in the six districts of Sikkim as of September 2023. During the same period, in July 2023, backyard cattle and free-ranging Yaks in the Gangtok district exhibited clinical signs of pyrexia, inappetence, and generalised round-raised nodules, indicative of an LSD outbreak.

### 2.5. Clinical Samples

Samples were collected from cattle (*n* = 9) raised in a backyard system and free-ranging yaks (*n* = 5) from the Gangtok district of Sikkim. The samples included nasal swabs, scab tissues, skin scrapings in a viral transport medium, and whole blood (2.5 mL) from animals displaying clinical signs. Additionally, vector samples, including flies (Stomoxys, *Musca domestica*, and mosquito) (*n* = 1) in the vicinity of the cattle, as well as the Hyalomma tick and Hypoderma fly (*n* = 1) from the yak, were also collected in methanol-containing bottles and transported to ICAR-NIVEDI, Bengaluru by maintaining proper cold chain, for laboratory diagnosis. Additionally, the blood samples (2.5 mL per animal) were collected from the jugular vein of apparently healthy cattle from the districts of Sikkim (Mangan, Gyalshing, and Soreng) for serological studies (Table 1).

### 2.6. Sample Processing

The scab tissue samples were triturated to create a 10% suspension in phosphate-buffered saline (PBS, pH 7.2), followed by filtration using a 0.45 μM syringe filter. The nasal swabs were suspended in 1 mL of PBS. The vector samples were processed similarly to the scab tissue, with the whole vector being triturated using a pestle and mortar to create a 10% suspension in PBS, followed by filtration with a 0.45 μM syringe filter. Following the manufacturer’s instructions, DNA extraction from these processed samples was carried out using the DNeasy Blood and Tissue Kit (Catalogue no. 69506, Qiagen, Germany). Briefly, 200 μL of the whole blood/nasal swab suspension/triturated scab tissue suspension/vector suspension was taken, lysis was performed for 20 min at 56 °C, and final elution was performed with elution buffer in 30 μL. The concentration of the DNA was assessed using a Nano spectrophotometer (Nabi), and the DNA was stored at −20 °C until further use. All the biological materials were disposed of after experiments as per standard operating procedures (SOPs) laid down by the Institute Bio-safety Committee (IBSC).

### 2.7. Molecular Identification of the Agent

The extracted DNA from the nasal swabs, scab tissues, whole blood, and vector samples was initially amplified for the *Capripoxvirus*-specific partial *P32* gene (237 bp) [20] and followed by LSDV-specific EEV glycoprotein gene- LSDV126 (122 bp) [21] using the respective forward and reverse primers. Additionally, the full-length genes, namely major envelope protein (*P32)*, G-protein coupled receptor (*GPCR)*, and RNA polymerase (*RPO30)* genes of LSDV, were amplified for confirmation and phylogenetic analysis. The sequences of the primers and their annealing temperatures used in the PCR assays are listed in Table 2.

The conventional PCR assay was performed using a thermal cycler (Bio-Rad, Hercules, CA, USA) with a total reaction volume of 25 μL. The reaction mixture was composed of 12.5 μL of PCR master mix (Dream *Taq* Green PCR Master mix 2x, Thermo Scientific), 1 μL each of forward and reverse primers (10 pmol/μL), 8.5 μL of nuclease-free water, and 2 μL of template DNA. DNA from the reference virus LSDV/Cattle/Chitradurga/P34 was used as a positive control. The non-template control consisted of nuclease-free water in place of the DNA template. The PCR products were analyzed by agarose gel electrophoresis on a 1.5% agarose gel in 1X TAE buffer. Fragments of the appropriate size were excised, and gel elution was performed using the NucleoSpin Gel and PCR Clean-up kit (Macherey-Nagel, Düren, Germany).

### 2.8. Sequence and Phylogenetic Analysis

The gel-purified PCR products of full-length *P32* (1012 bp), *GPCR* (1200 bp), and *RPO30* (840 bp) were subjected to sequencing using their respective forward and reverse primers (Table 2) by the Sanger sequencing method at Eurofins Genomics India Private Limited, Bengaluru, India. The sequenced data of *P32*, *GPCR*, and *RPO30* were analyzed by editing the sequences using the Gene tool (Informer Technologies, Inc.) to 969 bp, 1146 bp, and 606 bp, respectively. For comprehensive phylogenetic analysis, nucleotide sequences corresponding to putative genes (*P32*, GPCR, and *RPO30*) associated with Capripoxvirus, both originating from India and other countries, were retrieved from the GenBank database. These sequences were aligned by MUSCLE alignment in MEGA version 11 software and using the Tamura model with 1000 bootstrap values the maximum likelihood (ML) phylogenetic tree was constructed.

### 2.9. Virus Isolation

Vero cells maintained in 10% growth media (MEM with 10% FBS) were used for virus isolation. The filtered scab tissue suspension from Cattle (n = 1) and Yak (n = 1) were separately inoculated onto a confluent monolayer of Vero cells in a 25 cm² flask. The infected flasks were incubated at 37 °C in a 5% CO_2_ incubator and observed daily for the presence of cytopathic effects (CPE).

### 2.10. Indirect-Enzyme Linked Immuno Sorbent Assay (iELISA)

An in-house developed ELISA kit was used to determine the presence of antibodies against LSDV in the serum samples. The LSDV ORF117 recombinant protein prepared from the virus LSDV/Cattle/India/Chitradurga was used at a concentration of 50 ng per well as the coating antigen in carbonate bicarbonate buffer (pH 9.6). The plates were incubated overnight at 4 °C, washed thrice with wash buffer (PBS with 0.05% Tween 20), and blocked with 1% bovine gelatin in wash buffer for 1 h at 37 °C. After blocking, the plates were washed thrice, and the serum samples, as well as known positive and negative serum samples, diluted in wash buffer at a ratio of 1:150, were added to the plates and incubated for 1 h at 37 °C. Following this, rabbit anti-bovine IgG HRP conjugate (#A10-102P, Bethyl Laboratories) at a 1:7500 dilution in blocking buffer was added and incubated for 1 h at 37 °C. Detection involved using ortho phenylenediamine dihydrochloride (OPD, Sigma-Aldrich) and measuring the optical density at 492 nm with the help of an ELISA plate reader (Tecan Infinite F50). The percentage positivity (PPV) was obtained for each serum sample by calculating the ratio of the difference in the Optical Density (OD) value of the test serum and negative serum control to the difference in the OD value of the positive serum control and negative serum control. Test serum samples with a PPV value of more than 40% were considered positive.

### 2.11. Serum Neutralisation Test (SNT)

The serum neutralisation assay (SNT) was performed following a previously described protocol, albeit with minor modifications [22]. In brief, MDBK cells were grown in 96-well tissue culture plates to 90% confluency. All serum samples, including the test, known positive, and negative samples, were heat-inactivated at 56 °C for 30 min. Following that, the serum samples were two-fold serially diluted in MEM and incubated for 1 h at 37 °C with an equal volume of 100 TCID_50_ virus suspension, which was prepared using the reference virus LSDV/Cattle/India/Chitradurga/P34. Then, the virus-antibody mixture was added to the MDBK cells grown in the 96-well plate and observed daily for the development of characteristic cytopathic effect (CPE). The final readings of the plates were taken 96 h post-infection, and a titre of more than 1:8 was considered positive.

## 3. Results and Discussion

### 3.1. Epidemiological Investigation and Clinical Signs

**Figure 1:**
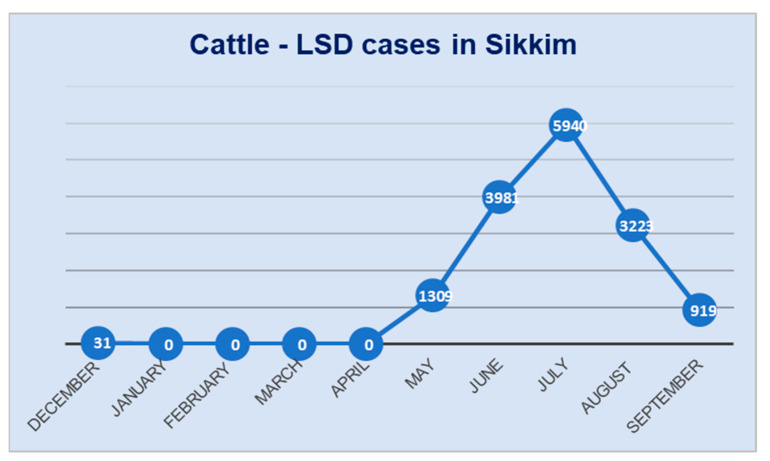
The line diagram showing the number of LSD cases recorded in cattle in the state of Sikkim during the years 2022–2023.

**Figure 2:**
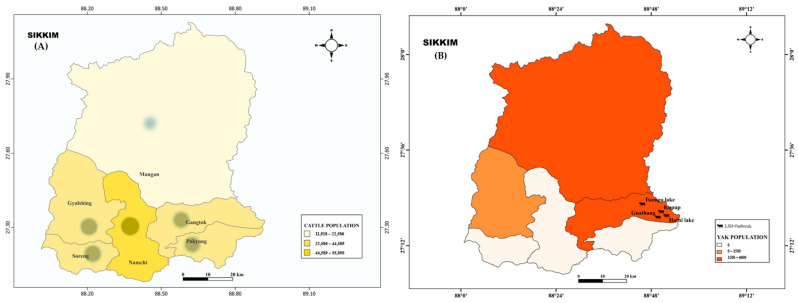
The population density maps display the outbreaks reported in (**A**) Cattle and (**B**) Yak species across the districts of Sikkim. In the cattle population map, the circle intensity indicates the number of attacks and the number of deaths that occurred in cattle in each district. The symbol 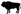 represents the emergence of LSD in Yak species reported in the Gangtok district of Sikkim.

The first confirmed case of LSD from cattle in Sikkim was reported in December 2022. Following this, no further cases were documented until April 2023. However, in May 2023, LSD outbreaks started to emerge and were reported in all six districts of Sikkim (Figure 1). As a full-blown LSD outbreak unfolded in cattle, suspected cases of LSD, akin to those seen in cattle, were also observed in yaks, particularly in the Gangtok district of Sikkim (Figure 2A,B).

**Figure 3:**
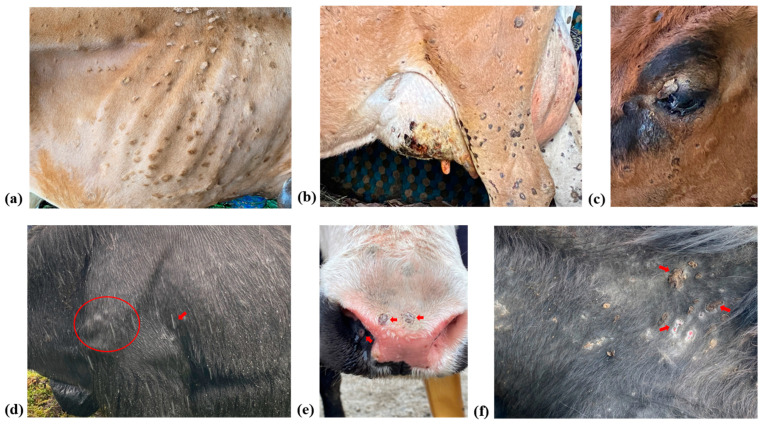
This figure illustrates the clinical signs observed in Cattle (**a**–**c**) and Yak (**d**–**f**), showcasing characteristic skin nodules in various regions. In cattle, nodules are visible on the (**a**) thoracic region, (**b**) hind limb and udder, and (**c**) upper eyelid. In yaks, nodules are evident on the (**d**) elbow joint, (**e**) muzzle and nasal mucosa, and (**f**) neck region.

During the peak of the LSD outbreaks, a detailed epidemiological investigation was carried out in the state of Sikkim in the affected regions. The cattle displayed clinical signs consistent with LSD, elevated body temperature, reduced milk yield in dairy cows, oedema of dependent body parts, and typical nodular skin lesions measuring 0.5–3 cm (Figure 3a–c). Similar varied clinical signs and lesions depending on the stage of the disease have also been reported from different parts of the world in cattle affected with LSDV [2,22]. The morbidity, mortality, and case fatality rates for cattle were 9.08%, 1.84%, and 20.24%, respectively. Even though the case fatality rates in LSD-affected cattle are typically reported to be within 10% in endemic areas [23,24], there have been instances of case fatality rates reaching up to 50% [25]. Since the disease had not been reported in this region previously, the increased case fatality rate observed in our study might be attributed to the lack of LSD vaccination as well as the presence of the naïve population.

Similarly, affected yaks exhibited typical LSD clinical signs (Figure 3d–f) with morbidity, mortality, and case fatality rates of 7.57%, 1.24%, and 16.33%, respectively. The mortality rate recorded in our study was similar to cattle. However, a higher mortality rate (53.33%) due to LSD has been recorded in yaks from China recently [4]. During the investigation, it was noted that mixed grazing and cattle movement occur in hilly regions during off-seasons, where yaks are also reared. Further, considering the presence of similar clinical patterns, morbidity, mortality, case fatality rate, and spatiotemporal distribution among affected cattle and yak, along with abundant vectors, suggest that the cross-species spillover of LSD from cattle to yak may be attributed to these factors.

### 3.2. Detection of LSDV and Serological Analysis

Diagnosis of LSD based on the clinical signs might be difficult as only a few skin lesions or transient fever may be confused with the skin lesions of other diseases such as pseudo-lumpy skin disease, bovine dermatophilosis, onchocerciasis, besnoitiosis, demodex infection, insect bite, and urticaria. Hence, for rapid and accurate laboratory diagnosis, molecular techniques such as PCR are often used as the primary tool [26]. In this study, DNA extracted from both blood and tissue (scab/nasal swabs) samples collected from both cattle and yak were used as template in amplification of Capripoxvirus genus-specific *P32* gene by PCR assay, a method that has been successfully used in previous studies for confirmation of the presence of Carpripoxvirus [20]. Subsequently, the samples were subjected to PCR amplification targeting the LSDV-specific EEV glycoprotein gene of amplicon size 122 bp, as it is sensitive and specific to LSDV field strains [21]. Out of nine cattle tested by both PCR assays, three were found to be positive, and six were found negative. Skin scabs are the most reliable samples to detect LSDV even after three months of infection [2,27]. All the blood samples from cattle were found negative, but yak blood samples were positive which indicates active viremia in yak as compared to cattle. Among the different clinical samples, the LSD viral DNA could be detected in the blood samples during the viraemic stage which lasts between 4–12 days [26]. Therefore, the time of collection of blood samples and the virus titre in the blood samples greatly affect the disease diagnosis [26]. Furthermore, four out of five yak samples tested were found to be positive for the Capripoxvirus-specific gene as well as the LSDV-specific EEV glycoprotein gene (Table 3).

LSD occurrence is attributed to transmission that is primarily seasonal and mainly associated with the abundance of arthropod vectors. However, it can occur at any time of the year, as no season is vector-free [22]. Vectors such as *Stomoxys calcitrans* (stable fly), *Biomya fasciata* (biting fly), *Musca domestica* (house fly), *Glossina* sp. (tsetse fly), *Culex* sp. and *Aedes aegypti* (mosquitoes), *Culicoides* sp. (biting midge), and ticks such as *Amblyomma* sp., *Rhipicephalus* sp., *Boophilus* sp., and *Hyalomma* sp. are involved in LSD transmission and spread of the disease [15,28]. In this study, the vectors collected from cattle and yak were identified as Stable fly (*Stomoxys* sp.), House fly (*Musca domestica*), mosquito, warble fly (*Hypoderma* sp.), and tick (*Hyalomma* sp.). All DNA samples extracted from these vectors tested positive for the Capripoxvirus-specific gene (*P32*) as well as the LSDV-specific gene (EEV glycoprotein), strongly suggesting that cross-species transmission between cattle and yak was mediated by these arthropod vectors.

Virus isolation was performed for confirmation, as it is considered the gold standard test for LSDV identification [29]. The Vero cell line, known for its ability to yield high virus titres was used for virus isolation. Scab tissue samples, one each from cattle and yak species, were subjected to isolation. After four blind passages, the cells exhibited characteristic cytopathic effect (CPE) as aggregation and rounding of cells, aligning with previous reports [30].

Out of four random apparently healthy serum samples collected from cattle in the affected zone, three were found positive for LSDV antibodies in ELISA and serum neutralisation test with titres ranging from 1:8 to 1:32. Similarly, five clinically healthy yaks were analyzed for serology from the affected herds, and all animals were found positive in both ELISA and SNT, indicating the presence of anti-LSDV antibodies. The presence of low antibody titres in serum samples might be attributed to mild infection [31]. Additionally, serum samples (n = 36) collected from apparently healthy cattle from neighbouring districts of Sikkim (Mangan, Soreng, and Gyalshing) showed overall seropositivity of 13.9%, aligning with other studies [32]. However, this is in contrast to previous reports [33,34], which could be due to differences in exposure to LSDV, vector population, cattle density, the immune status of the animals, time of sample collection, and sample size.

### 3.3. Sequence and Phylogenetic Analysis

**Figure 4:**
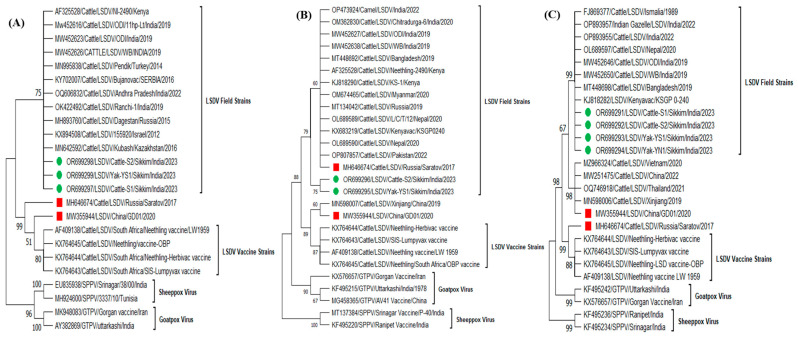
Phylogenetic analysis of full-length genes (**A**) *P32*, (**B**) *RPO30*, and (**C**) GPCR sequences of LSDV from Cattle and Yak. The phylogenetic tree is constructed based on the virus isolated from cattle and yak during the Sikkim LSD outbreak, in comparison to reference strains from GenBank. The tree was constructed using the maximum likelihood method with 1000 bootstrap values in Molecular Evolutionary Genetics Analysis (MEGA) Version.11 software (http://www.megasoftware.net). Both isolates from Cattle and Yak species are clustered with LSDV field strains circulating in India. The round (Green) symbols indicate the newly obtained isolates in this study, while the square (Red) symbols indicate the recombinant strains of LSDV.

To further, confirm the genetic makeup of the LSDV in Yak, the LSDV isolates (LSDV/Cattle/S1/Sikkim/India/2023, LSDV/Cattle/S2/Sikkim/India/2023, LSDV/Yak/YS1/Sikkim/India/2023 and LSDV/Yak/YN1/Sikkim/India/2023) from both cattle and yak were sequenced and phylogenetic analysis based on *P32*, *RPO30,* and GPCR gene sequences was carried out. The sequences were edited and submitted to GenBank and received accession numbers [OR699297, OR699298 (*P32*), OR699296 (*RPO30*) and OR699291, OR699292 (GPCR) for cattle LSDV isolates and OR699299 (*P32*), OR699295 (*RPO30*) and OR699291, OR699292 (GPCR) for Yak LSDV isolates]. The sequence analysis revealed that the respective genes (*P32*, *RPO30,* and GPCR) of LSDV isolates from cattle and yak showed 100% similarity among themselves, indicating that the spillover of LSDV from cattle appears to have caused the disease in yaks. The isolates from this study clustered with field strains circulating in various regions, including India, Kenya (KSGP 0240, KS-1, NI-2490), Turkey, Russia, Bangladesh, and other neighbouring states of India (Figure 4). The isolates from cattle and yak in this study closely resembled the virus isolates circulating in the Indian sub-continent, which are closely related to the South African Kenyan LSDV isolates [2]. The genetic similarity between LSDV isolates from cattle and yak was supported by the observation of similar clinical and epidemiological patterns of the disease in both species during the investigation. This indicated that the same strain of LSDV was affecting both cattle and yak. However, it is interesting to note that the situation in China is different, where a report suggests that yak experienced higher mortality rates than cattle due to a recombinant strain of LSDV [4]. In contrast, in India, this study reports the first occurrence of the disease in yak due to the spillover of a field strain of LSDV from cattle, not a recombinant strain.

**Figure 5:**
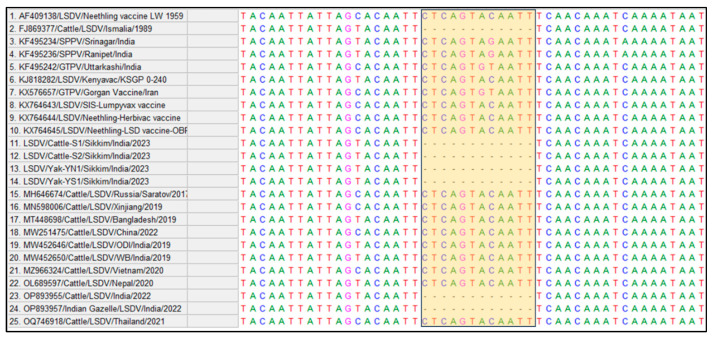
Multiple sequence alignment of GPCR sequences of Cattle and Yak LSDV isolates along with other Capripoxviruses circulating worldwide, showing the 12-nucleotide deletion (94–105) (highlighted region).

The sequence alignment of GPCR genes from LSDV isolates in this study and other circulating strains revealed a 12-nucleotide deletion (Figure 5). The strength of nucleotide deletion is a unique feature of LSD isolates from recent LSD outbreaks in India as compared to the first LSD outbreak report in 2019 in India [2]. This deletion has been previously identified in LSDV field strains from the Middle East, Europe, and Balkan countries [16]. The presence of this 12-nucleotide deletion (94–105) in the GPCR gene in the recent outbreak of LSDV isolates (2022–2023) could be used as a genetic marker for differentiation of past field LSDV isolates (2019) in India [2]. Further, the phylogenetic analysis of the *RPO30* gene supports this distinction, as the isolates from the present study formed a separate sub-group that clustered with LSDV field strains but deviated from the main branch. This deviation suggests the emergence of LSD in atypical hosts from cattle, demonstrating the virus’s adaptability and pathogenicity across different species.

For better prevention and control of LSD, vaccination in combination with good farm-level biosecurity measures are advised [35]. Globally, both heterologous (sheep pox and goat pox) and homologous vaccines (LSD) are being used to prevent and control the disease. Currently, at least twelve LSD vaccines for cattle have been described and are being used in different parts of the world for immunisation [35] including the recently developed vaccine from India [36]. As the yak species is closely related to cattle, the same vaccination protocol may be used until further studies.

In conclusion, the significant LSD outbreaks in Sikkim that have impacted the cattle population, the close interaction of the yak population with the infected animals, and the potential presence of vectors collectively lead to the conclusion that this study marks the first documented occurrence of LSD in the new host species, Yak, in India as a spillover event. Yak is a robust and hardy bovine species native to high-altitude regions of the North-Eastern part of India and other Himalayan and Central Asian countries. In view of the vulnerable status of these Yaks according to the International Union for Conservation of Nature (IUCN), it is crucial to design a strategy for the prevention and control of LSD in these species.

## Figures and Tables

**Table 1:** Samples collected from Cattle and Yak species from the districts of Sikkim.

State	District	Species	Type of Sample					
			Scab Tissue (*n*)	Skin Scraping (*n*)	Nasal Swabs (*n*)	Whole Blood (*n*)	Serum (*n*)	Vector (*n*)
**Sikkim**	Gangtok	Cattle	2	2	3	4	4	3
	Mangan	Cattle	-	-	-	-	12	-
	Soreng	Cattle	-	-	-	-	12	-
	Gyalshing	Cattle	-	-	-	-	12	-
	Gangtok	Yak	4	1	1	5	5	2

**Table 2:** Primers used for conventional PCR assays targeting various genes of LSDV.

Gene	Primer	Sequence (5′-3′)	Annealing Temp. & Time	Amplicon Size
**Partial *P32***	SGPP 32-F	ACACAGGGGGATATGATTTTACC	52 °C, 30 sec	237 bp
	SGPP 32-R	ATACCGTTTTTCATTTCGTTAGC		
***LSD126***	LSDV-F	TAGAAAATGGATGTACCACAAATACAG	60 °C, 1 min	122 bp
	LSDV-R	TTGTTACAACTCAAATCGTTAGGTG		
**Full-length *P32***	B7-F	AACACTCTCATTGGTGTTCGG	56 °C, 1 min	1012 bp
	A95-R	CACATGGCAGATATCCCATTA		
***GPCR***	GPCR- F	TTTATCAGCACTAGGTCATTATCT	59 °C, 1 min	1200 bp
	GPCR- R	TATCACTCCCTTCCATTTTTAT		
***RPO30***	*RPO30*-F	ATAACCTACATGCATAAACAGAAG	52 °C, 1 min	840 bp
	*RPO30*-R	ATACGAATCTACTTCATCACAAGA		

**Table 3:** Details of the samples collected and PCR results.

Sl. No.	Species	Type of Sample	PCR Result		Virus Isolation
			*P32* Gene	EEV Gene	
1.	Cattle (JCB)	Scab/Nasal swab	Positive	Positive	LSDV/Cattle/S1/Sikkim/India LSDV/Cattle/S2/Sikkim/India
2.	Cattle (JCB)	Scab/Nasal swab	Positive	Positive	
3.	Cattle (JCB)	Nasal Swab	Positive	Positive	
4.	Cattle (HF CB)	Blood	Negative	Negative	
5.	Cattle (JCB)	Blood	Negative	Negative	
6.	Cattle (JCB)	Blood	Negative	Negative	
7.	Cattle (HF CB)	Blood	Negative	Negative	
8.	Cattle (JCB)	Skin biopsy	Negative	Negative	
9.	Cattle (HF CB)	Skin biopsy	Negative	Negative	
1.	Yak	Scab/Nasal swab/Blood	Positive	Positive	LSDV/Yak/YS1/Sikkim/India LSDV/Yak/YN1/Sikkim/India
2.	Yak	Scab/Blood	Positive	Positive	
3.	Yak	Skin biopsy/Blood	Negative	Negative	
4.	Yak	Scab/Blood	Positive	Positive	
5.	Yak	Scab/Blood	Positive	Positive	

JCB = Jersry Cross breed; HF CB = Holstein Friesian Cross breed.

## Data Availability

The data presented in this study are available on request from the corresponding author. The data are not publicly available due to the laid down rules of the institute.
